# Creatine supplementation is safe, beneficial throughout the lifespan, and should not be restricted

**DOI:** 10.3389/fnut.2025.1578564

**Published:** 2025-04-04

**Authors:** Richard B. Kreider, Andrew R. Jagim, Jose Antonio, Douglas S. Kalman, Chad M. Kerksick, Jeffrey R. Stout, Robert Wildman, Rick Collins, Diego A. Bonilla

**Affiliations:** ^1^Exercise and Sport Nutrition Laboratory, Human Clinical Research Facility, Texas A&M University, College Station, TX, United States; ^2^Sports Medicine, Mayo Clinic Health System, La Crosse, WI, United States; ^3^Exercise and Sport Science Department, University of Wisconsin—La Crosse, La Crosse, WI, United States; ^4^Patriot Performance Laboratory, Frank Pettrone Center for Sports Performance, Intercollegiate Athletics, George Mason University, Fairfax, VA, United States; ^5^Department of Health and Human Performance, Nova Southeastern University, Davie, FL, United States; ^6^Dr. Kiran C. Patel College of Osteopathic Medicine, Nova Southeastern University, Davie, FL, United States; ^7^Exercise and Performance Nutrition Laboratory, College of Science, Technology, and Health, Lindenwood University, St. Charles, MO, United States; ^8^Physiology of Work and Exercise Response (POWER) Laboratory, Institute of Exercise Physiology and Rehabilitation Science, University of Central Florida, Orlando, FL, United States; ^9^Department of Human Nutrition, Kansas State University, Manhattan, KS, United States; ^10^Collins Gann McCloskey and Barry PLLC, Mineola, NY, United States; ^11^Research Division, Dynamical Business and Science Society–DBSS International SAS, Bogotá, Colombia; ^12^Hologenomiks Research Group, Department of Genetics, Physical Anthropology and Animal Physiology, University of the Basque Country (UPV/EHU), Leioa, Spain; ^13^Grupo de Investigación NUTRAL, Facultad Ciencias de la Nutrición y los Alimentos, Universidad CES, Medellín, Colombia

**Keywords:** dietary supplements, creatine monohydrate, adolescent nutritional physiological phenomena, frail older adults, transition to adult care, public policy, nutritional epidemiology

## Introduction

As researchers investigating creatine supplementation, we have become increasingly concerned about reports that government agencies are attempting to restrict the sale of dietary supplements, including dietary supplements containing creatine, to children and adolescents. Creatine is a naturally occurring compound found in every cell in the human body that plays a critical role in cellular metabolism. The daily turnover of creatine is about 2–4 grams/day, depending on muscle mass and physical activity levels (1, 2). About half of the daily need for creatine is synthesized in the body from amino acids (arginine, glycine, methionine) and stored as free creatine or phosphocreatine in muscle, brain, heart, and other tissues (1). The remaining daily need to maintain normal cell and tissue levels of creatine primarily comes from consuming meat and fish. For example, one pound (16 oz.) of red meat and fish contains about 1–2 grams of creatine. In the cells, creatine changes into phosphocreatine, a compound vital in maintaining cellular energy availability, particularly during metabolically stressful conditions like intense exercise, periods of injury or illness, and some metabolic diseases with applications for diverse populations across a wide age range.

**Figure 1:**
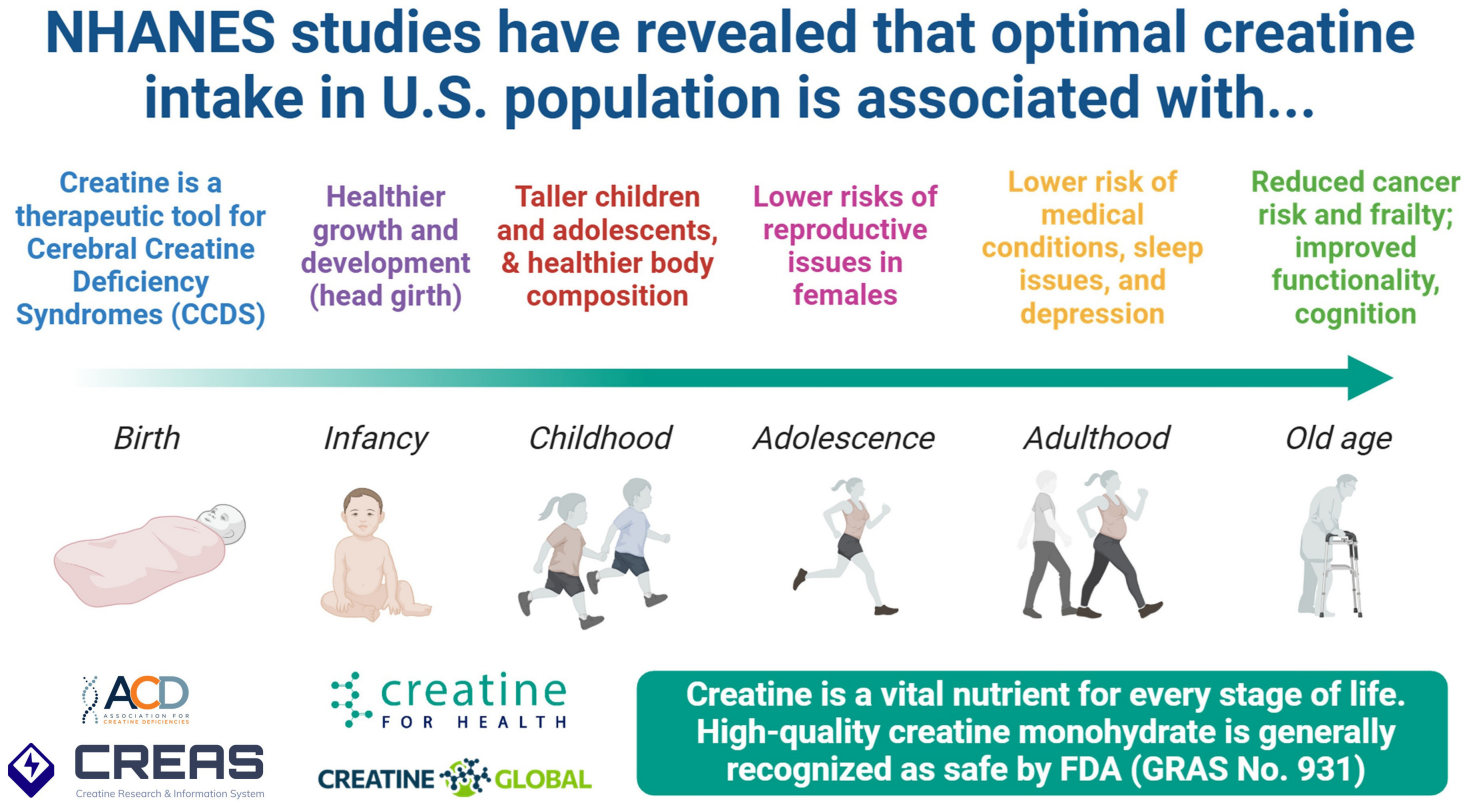
Creatine is an important nutrient throughout the lifespan. Readers are encouraged to visit the Association for Creatine Deficiencies (ACD) for more information about Cerebral Creatine Deficiency Syndromes (CCDS)—available at https://creatineinfo.org/, as well as Creatine For Health to know more about the research-based information for educational purposes to promote awareness of the importance of creatine in health and disease—available at https://creatineforhealth.com/, and the Creatine REsearch And information System (CREAS), a DBSS project that implements bibliometrics, scientometrics, and other AI-assisted analyses to routinely inform, develop, improve and support research endeavors and clinical practice (available at: https://creas.pro/). Created with BioRender.com (DB).

Creatine is essential to promote normal energy metabolism and healthy growth and maturation in children and adolescents (Figure 1). Low dietary creatine intake has been associated with slower growth, less muscle mass, and higher body fat in children and adolescents (3). Adolescents have been reported to consume lower than recommended amounts of creatine in the diet. Despite common misconceptions, creatine has a well-supported safety profile and has been repeatedly shown to be safe, even with long-term supplementation (4, 5). Additionally, there is no evidence that children and adolescents purchasing and taking creatine-containing supplements cause adverse health effects and/or increase the likelihood of eating disorders or use of performance-enhancing drugs. Conversely, individuals who take creatine are interested in improving health, exercise performance, gaining muscle mass, and improving their physique. While meat and fish are natural sources of creatine, they can be expensive and high in calories. A food-first approach is always recommended, meaning that dietary sources of creatine should be prioritized whenever possible. However, due to cost, accessibility, and other potential barriers, dietary supplementation of creatine monohydrate or supplements and foods fortified with creatine monohydrate are a cost-effective way to ensure that children and adolescents obtain enough creatine in their diet to promote healthy growth and maturation. Creatine supplementation has also been shown to be safe and have clinically meaningful benefits in pediatric disorders, including acute lymphoblastic leukemia, Duchenne muscular dystrophy, and disorders of creatine metabolism.

Legislation restricting the sale of creatine-containing products to children and adolescents is not based on scientific evidence which strongly supports the importance of creatine in the diet and its safety as a supplement. Moreover, creatine supplementation is not associated with eating disorders (6), and any claim suggesting the contrary is not rooted in scientific evidence. These false claims and reckless speculation regarding the dangers of creatine supplementation may discourage the use of creatine by minors, parents of minors, and healthcare professionals from recommending creatine supplementation, a nutrient that offers a plethora of health and performance-related benefits for all populations. This may further reduce the availability of creatine in children's and adolescents' diets, impairing growth and maturation and negatively impacting the development of a healthy body composition. Lobbying groups and legislatures should base laws on the available science, not speculation, unfounded hypotheses, or politics. We provide the following scientific facts about creatine to help those proposing legislative efforts to limit the availability of creatine in children and adolescents make more informed legislation.

## Creatine facts

1. Creatine is a naturally occurring compound that is a primary constituent of phosphocreatine stored in cells and is needed to provide cellular energy.
2. The daily need for creatine is about 2–4 grams/day, depending on muscle mass and physical activity levels. About half of the daily need for creatine is synthesized in the body from the amino acids arginine, glycine, and methionine. The remainder must be obtained from the diet and/or dietary supplements.
3. The best sources of creatine in the diet are meat and fish, which contain about 1–2 grams of creatine per pound. Since meat and fish are expensive (about $4.00 to $18.00 USD per pound) and contain large amounts of protein and fat (i.e., about 450 to 1,400 kcals/pound), dietary supplementation of creatine monohydrate (about $0.03–$0.05 USD per gram) is a more cost-effective way to ensure individuals obtain enough creatine in their diet to meet daily needs (1).
4. Creatine supplementation can also be an effective dietary strategy for vegans or vegetarians who often do not consume enough creatine in their diet (7).
5. Analysis of the National Health and Nutrition Examination Survey (NHANES) database revealed that 4,291 boys and girls aged 2–19 years (3) consumed an average of 1 gram/day of creatine in their diets, and higher dietary intake of creatine (>1.5 grams/day) was associated with greater height and weight compared to those consuming diets lower in creatine. Dietary creatine intake was also positively correlated with lean mass and bone mineral content while negatively correlating with fat mass and body fat percentage in 1,273 children and adolescents between the ages of 8 and 19 years (8). These findings indicate that the dietary availability of creatine in children and adolescents may positively affect growth, maturation, and body composition. Yet, in recent years, younger populations have been reported to have decreased dietary creatine intake (9), underscoring the need for children and adolescents to consume more creatine in their diets.
6. The adequate intake (AI) for creatine is 7 mg/day for infants aged 0–6 months who are exclusively breastfed and 8.4 mg/day for infants aged 7–12 months (10).
7. The NHANES database also revealed that lower dietary creatine intake (i.e., <0.95 grams/day) was associated with poorer cognitive function test performance among 1,340 adults ≥60 years compared to those consuming diets with >0.95 grams per day (11). Additionally, analysis of dietary creatine intake among 1,500 adults ≥65 years revealed that 70% of this cohort consumed less than recommended amounts of creatine in their diets (<0.95 grams per day), and low dietary creatine intake was associated with a greater risk of angina pectoris and liver conditions compared to those consuming >1.0 grams per day of dietary creatine (12). These findings highlight the need for older individuals to increase dietary intake of creatine.
8. High-quality creatine monohydrate is Generally Recognized as Safe (GRAS) by the Food and Drug Administration (13) and is considered safe for human consumption in dietary supplements in the United States, Canada, Europe, Australia, South Korea, Japan, and China. Efforts are underway to fortify creatine in food and to position it as a conditionally essential nutrient.
9. Over 680 peer-reviewed clinical trials have been conducted on creatine supplementation (95% as creatine monohydrate) since the 1970s, involving over 12,800 study participants administered creatine supplements in dosages up to 30 grams per day for 14 years in populations ranging from infants to very elderly individuals in both healthy and clinical populations. No clinical adverse events were reported in any clinical trial study, and the minor side effects reported were infrequent and not significantly different from over 13,500 participants consuming placebos in these studies. This includes a comparison of studies conducted on children and adolescents (<18 years), young adults (19–45 years), middle-aged adults (46–65 years), and older adults (>65 years). Moreover, an analysis of over 28.4 million adverse event reports in the United States, Canada, Australia, and Europe, using SIDER 4.1 over the last 50 years, reveals that creatine has rarely been mentioned (about 0.0007%) despite billions of doses taken worldwide over the past 30 years. While adverse event reports do not imply causality, the lack of reports worldwide supports findings from clinical trials that creatine is safe for individuals of all ages.
10. Creatine monohydrate supplementation (e.g., 0.3 grams/kg/day for 5–7 days and 0.05 to 0.15 grams/kg/day thereafter) is the most effective nutritional strategy to increase and maintain tissue creatine content (1). Many studies indicate that creatine monohydrate supplementation increases gains in strength, high-intensity exercise performance, and muscle mass during resistance-exercise training (5, 14). It is considered the most effective nutritional strategy for individuals wanting to maintain and increase strength (5). Creatine supplementation has also been reported to reduce the risk of injury, including the severity of concussion and traumatic brain injury (2). Restricting the availability of creatine to children and adolescents may put them at risk for injury or compromise recovery following injury or disease management for neurocognitive disorders.
11. Emerging evidence indicates that creatine monohydrate supplementation possesses a number of health benefits during pregnancy and infancy (15), for children and adolescents (16), for women (17), for adults involved in exercise training (5), and for older populations (18). Additionally, there is evidence that creatine monohydrate supplementation enhances immunity (19) and can promote heart (20), vascular (21), and brain health (22). Therapeutic benefits have been reported in the management of diabetes (23), sarcopenia (24–27), osteoporosis (25, 28), patients with neuromuscular diseases (29), and rehabilitation (4, 24, 30–36). Furthermore, data shows that creatine slows the progression of some forms of cancer (37, 38) and may have therapeutic benefit in helping cancer patients maintain muscle mass (39) and prevent body fat accumulation during maintenance chemotherapy that includes corticosteroids (40). For this reason, it is recommended that all individuals consume 2–3 grams per day of creatine to promote general health (2, 5, 41).
12. Several studies, particularly in older populations, have shown that consuming diets higher in creatine (>0.95 grams/day) is associated with better cognition (6) and that creatine supplementation may improve cognitive function (42–45).
13. No evidence is available to demonstrate that consuming creatine monohydrate increases the prevalence of eating disorders or adversely affects individuals being treated for psychiatric conditions (6). Conversely, analysis of the NHANES database among 22,692 adults indicates that low dietary creatine intake is associated with a greater incidence of depression (45), which is often related to eating disorders and/or poor body image perceptions (46). Furthermore, creatine supplementation has been suggested as a potential nutritional adjunctive strategy to help manage depression and reduce suicidal ideations in individuals unresponsive to some psychiatric medications (47).

In summary, the robust body of evidence supports the safety and multifaceted benefits of creatine supplementation across all age groups. We urge lobbyists, policymakers, and health agencies to consult with leading creatine scientists, and to consider the full spectrum of scientific data before implementing restrictions that would have adverse public health and performance implications. This opinion letter was endorsed by leading creatine scholars (Table 1).

**Table 1:** ISSN members, international creatine researchers, and scholars supporting this statement.

**Name**	**Position**	**Institution**
Richard B. Kreider, PhD, FACSM, FISSN, FACN, FNAK	Professor and Director, Exercise & Sport Nutrition Lab	Department of Kinesiology and Sports Management, Texas A&M University, College Station, TX, United States
Andrew R. Jagim, PhD, CSCS^*^D, CISSN	Director of Sports Medicine Research	Mayo Clinic Health System, La Crosse, WI, United States
Jose Antonio, PhD, FISSN, FNSCA	Professor	Exercise and Sport Science, Nova Southeastern University, Davie, FL, United States
Michael J. Ormsbee, PhD, FACSM, FISSN, FNAK, CISSN, CSCS^*^D	Professor and Director	Institute of Sports Sciences & Medicine, Florida State University, Tallahassee, FL, United States
Chad M. Kerksick, PhD, FACSM, FNSCA, FISSN, CISSN, CSCS^*^D	Assistant Dean & Professor, Director, Exercise and Performance Nutrition Lab	Lindenwood University, St. Charles, MO, United States
Douglas S. Kalman, PhD, RD, CCRC, FACN, FISSN	Clinical Associate Professor - Nutrition	Dr. Kiran C. Patel College of Osteopathic Medicine, Nova Southeastern University, Davie, FL, United States
Diego A. Bonilla, ISAK 3	Scientific Director & Researcher	DBSS Research Division – Dynamical Business & Science Society; NUTRAL Research Group, Universidad CES, Medellin, Colombia
Jeffrey R. Stout, PhD, FNAK, FNSCA, FACSM, FISSN	Professor & School Director	School of Kinesiology and Rehabilitation Sciences, University of Central Florida, United States
Maurizio Balestrino, MD	Associate Professor of Neurology	Department of Neuroscience, Rehabilitation, Ophthalmology, Genetics and Maternal-Child Sciences (DINOGMI), University of Genoa, Italy
Scott C. Forbes, PhD, CSEP-CEP, FISSN	Associate Professor	Department of Physical Education Studies, Brandon University, Canada
Susan M. Kleiner, PhD, RD, CNS-E, FACN, FISSN	Owner/Founder	High Performance Nutrition LLC, Mercer Island, WA, United States
Ann Frost Brown, PhD, CISSN	Associate Professor & Associate Dean	Department of Movement Sciences, University of Idaho, United States
Sergej M. Ostojic, MD, PhD	Professor	Department of Nutrition and Public Health, University of Agder, Norway
Drew E. Gonzalez, PhD, CISSN, CSCSD, TSAC-FD, SCCC	Research Associate	Exercise & Sport Nutrition Lab, Texas A&M University, College Station, TX, United States
Bill I. Campbell, PhD, CSCS, FISSN	Professor of Exercise Science	University of South Florida, Tampa, FL, United States
Eric S. Rawson, PhD, CSCS, FACM	Professor	Health, Nutrition, and Exercise Science, Messiah University, Mechanicsburg, PA, United States
Tim N. Ziegenfuss, PhD, CSCS, FISSN	CEO	The Center for Applied Health Sciences, Canfield, OH, United States
Mark A. Tarnopolsky, MD, PhD, FRCP(C)	Professor of Pediatrics	McMaster Children's Hospital, Hamilton, Ontario, Canada
Darren G. Candow, PhD, CSEP-CEP, FISSN	Professor	Faculty of Kinesiology and Health Studies, University of Regina, Regina, SK, Canada
Abbie E. Smith-Ryan, PhD, FACSM, FNSCA, FISSN	Professor & Associate Chair for Research	Department of Exercise & Sport Science/Nutrition, University of North Carolina, Chapel Hill, NC, United States
Stacey Ellery, BBioMedSc (Hons), PhD	NHMRC Emerging Leader Fellow (EL2), Head Bioenergetics in Reproduction Group	The Ritchie Centre, Hudson Institute of Medical Research, Monash University, Clayton, VIC, Australia
Ralf Jäger, PhD, MBA, FISSN	Founder	Increnovo LLC, Whitefish Bay, WI, United States
Robert Wildman, PhD, RD, LD, CISSN	Research Associate	Department of Human Nutrition, Kansas State University, Manhattan, KS, United States
Craig Sale, PhD, FACSM	Professor of Human Physiology and Nutrition	Institute of Sport, Manchester Metropolitan University, Manchester, United Kingdom
Rick Collins, Esq., FISSN, NSCA-CSCS	Partner	Collins Gann McCloskey & Barry PLLC, Mineola, NY, United States
Michael D. Roberts, PhD	Auburn University Endowed Alumni Professor	School of Kinesiology, Auburn University, Auburn, AL, United States
Vargas-Molina Salvador, PhD	Researcher	Physical Education and Sport, Faculty of Medicine, University of Málaga, Spain; DBSS Research Division – Dynamical Business & Science Society
Susan J. Hewlings, PhD, RD	Sr Vice President of Scientific Affairs	Radicle Science, Cudjoe Key, FL, United States
Lem W. Taylor, PhD, FACSM, FISSN	Professor	Human Performance Lab, University of Mary Hardin-Baylor, Belton, TX, United States
Hamilton Roschel, PhD, RD	Associate Professor, Scientific/Executive Director	Center of Lifestyle Medicine, University of Sao Paulo, Sao Paulo, Brazil
Wagner Domingues, PhD, RD	Associate Professor	Research Group on Physical Activity and Rehabilitation for Special Groups, Air Force University, Rio de Janeiro, RJ, Brazil
Jeff Volek, PhD	Professor	Department of Human Sciences, The Ohio State University, Columbus, OH, United States
Bruno Gualano, PhD	Associate Professor	Center of Lifestyle Medicine, University of Sao Paulo, Sao Paulo, Brazil
Elfego Galvan, PhD, RD	Medical Student (MS-III)	A.T. Still University School of Osteopathic Medicine - Arizona, Mesa, AZ, United States
Philip D. Chilibeck, PhD, CSEP-CEP	Professor	College of Kinesiology, University of Saskatchewan, Saskatoon, SK, Canada
Jay R. Hoffman, PhD	Professor and Director	Sport Science Program, Ariel University, Ariel, Israel
Nicholas D. Barringer, PhD, RDN, CSSD, CSCS	Chief Academic Officer and Dean Graduate Studies	Lionel University, Carpinteria, California, United States
